# Genome-wide association studies on coronary artery disease: A systematic review and implications for populations of different ancestries

**DOI:** 10.1371/journal.pone.0294341

**Published:** 2023-11-29

**Authors:** Sarah Silva, Dorothea Nitsch, Segun Fatumo

**Affiliations:** 1 Department of Non-Communicable Disease Epidemiology, London School of Hygiene and Tropical Medicine, London, United Kingdom; 2 The African Computational Genomics (TACG) Research Group, MRC/UVRI, and LSHTM, Entebbe, Uganda; Penn State: The Pennsylvania State University, UNITED STATES

## Abstract

**Background:**

Cardiovascular diseases are some of the leading causes of death worldwide, with coronary artery disease leading as one of the primary causes of mortality in both the developing and developed worlds. Despite its prevalence, there is a disproportionately small number of studies conducted in populations of non-European ancestry, with the limited sample sizes of such studies further restricting the power and generalizability of respective findings. This research aimed at understanding the differences in the genetic architecture of coronary artery disease (CAD) in populations of diverse ancestries in order to contribute towards the understanding of the pathophysiology of coronary artery disease.

**Methods:**

We performed a systematic review on the 6^th^ of October, 2022 summarizing genome-wide association studies on coronary artery disease, while employing the GWAS Catalog as an independent database to support the search. We developed a framework to assess the methodological quality of each study. We extracted and grouped associated single nucleotide polymorphisms and genes according to ancestry groups of participants.

**Results:**

We identified 3100 studies, of which, 36 relevant studies were included in this research. Three of the studies that were included were not listed in the GWAS Catalog, highlighting the value of conducting an independent search alongside established databases in order to ensure the full research landscape has been captured. 743,919 CAD case participants from 25 different countries were analysed, with 61% of the studies identified in this research conducted in populations of European ancestry. No studies investigated populations of Africans living in continental Africa or admixed American ancestry groups besides African-Americans, while limited sample sizes were included of population groups besides Europeans and East Asians. This observed disproportionate population representation highlights the gaps in the literature, which limits our ability to understand coronary artery disease as a global disease. 71 genetic loci were identified to be associated with coronary artery disease in more than one article, with ancestry-specific genetic loci identified in each respective population group which were not detected in studies of other ancestries.

**Conclusions:**

Although the replication and validation of these variants are still warranted, these finding are indicative of the value of including diverse ancestry populations in GWAS reference panels, as a more comprehensive understanding of the genetic architecture and pathophysiology of CAD can be achieved.

## Introduction

Coronary artery disease (CAD) is the leading cause of death worldwide, characterized by the build-up of atherosclerotic plaque in the arteries which supply oxygenated blood to the heart [1–3]. Over the past decade, age-adjusted cardiovascular death rates have been decreasing in high-income countries, whilst the opposite is being observed in low- and middle-income countries with a substantial increase in death rates due to cardiovascular diseases [4]. Although there is a general understanding of the modifiable risk factors for CAD, between ancestries there is an evident disproportionality in the frequency of these CAD risk factors, and consequently the observed prevalence and severity of the disease [5]. Moreover, there may be risk factors for CAD in low- and middle-income settings that have not been observed in high-income settings, whereby the pathobiology of CAD may be different in contexts with additional yet-undefined risk factors. This has prompted efforts in the discovery of population-specific genetic drivers of CAD, which in combination with findings from causal risk factors of CAD, may reveal previously overlooked or otherwise unidentified opportunities to improve public health efforts in reducing the burden of CAD globally.

In an effort to better understand the association between genetic loci and diseases of interest, genome-wide association studies (GWAS) have evolved over the past decade to what is now the most popular approach [6]. GWAS have since gone on to transform our understanding of the genetic architecture of CAD, with over 241 independently associated variants at 198 loci identified to be associated with the disease to date, and more thought to be undetected as of yet [7–9]. Recent studies of CAD prevalence in diverse ancestry populations have suggested that some populations are more susceptible to CAD risk than others [10–12].

The GWAS Catalog is a notable database of GWAS studies, developed in collaboration by the National Human Genome Research Institute and EMBL’s European Bioinformatics Institute. The Catalog was established in an effort to provide a single reliable, searchable and freely available database of associations between known single nucleotide polymorphisms (SNPs) and complex diseases [13].

The aim of this study was to undertake a systematic review of GWAS on CAD, in order to summarize and describe what is known to date with regards to genetic variations associated with CAD for populations of different ancestries. This was achieved through conducting a systematic review on GWAS studies on CAD, with a focus on ancestry-specific findings.

## Methods

This systematic review was conducted in accordance with the Preferred Reporting Items for Systematic Reviews and Meta-Analyses (PRISMA) guidelines. The protocol of the systematic review was registered with the International Prospective Register of Systematic Reviews (PROSPERO) on 25^th^ August 2021, with the registration ID: CRD42021272726.

### Search strategy

**Fig 1:**
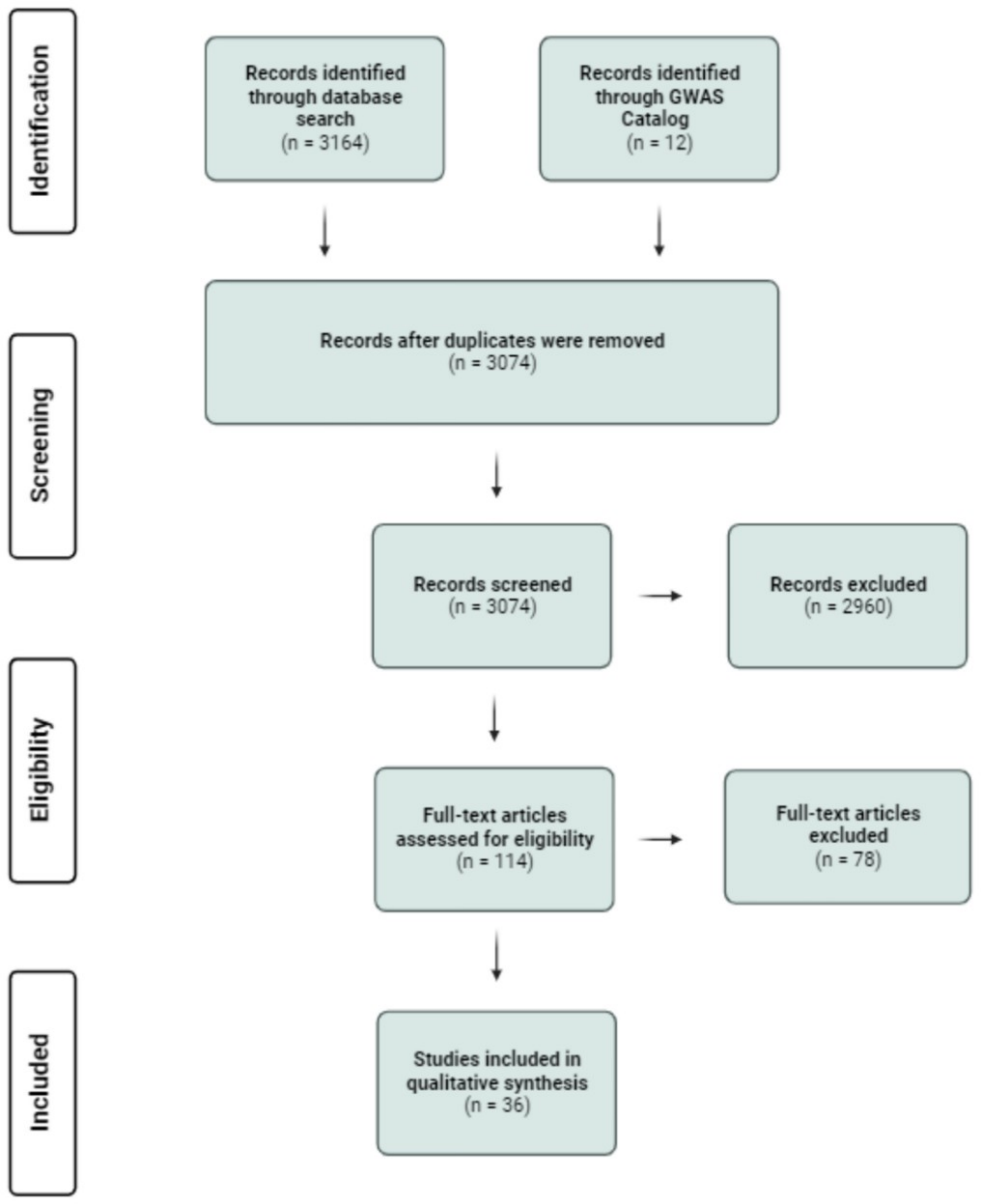
Preferred reporting items for systematic reviews and meta-analyses flowchart for the systematic review of genome-wide association studies of coronary artery disease.

A search was conducted in the databases Embase, Medline, Global Health, Web of Science and Cochrane Library on the 6^th^ of October, 2022. The terms “coronary artery disease”, “CAD”, “coronary heart disease”, “genome-wide association study” and “GWAS” were used in the search string, with a full record of the respective search strategies employed for the review shown in Fig 1 in S1 File.

The GWAS Catalog served as a reference when testing and developing the search strategy, and subsequently as an independent database in order to ensure that the research included all known GWAS papers related to CAD. A full list of studies listed in the GWAS Catalog associated with the trait “coronary artery disease” and included “coronary artery disease” as either a trait or background trait were additionally extracted. This list was compared to findings of the initial search in order evaluate how many studies were included in the Catalog that were picked up in this search, as well as if the Catalog had potentially overlooked other relevant studies.

### Eligibility screening

Studies were included if: (i) studies were published between 2005–2022, (ii) studies were published in the English language, (iii) studies included only human subjects, (iv) coronary artery disease was the primary outcome being investigated and (v) the study conducted a hypothesis-free GWAS.

The exclusion criteria of this review constituted of: (i) systematic reviews, (ii) non-human studies, (iii) studies without coronary artery disease as a primary outcome (iv) studies which had a hypothesis on which specific genes relate to CAD, (v) studies that only replicated previous findings, (vi) studies which didn’t employ GWAS as part of the methodology, and (vii) studies which restricted the study populations to participants who were diagnosed with other underlying diseases, such as diabetes, as opposed to representing CAD cases/controls from the general population in a given setting.

### Data extraction

The following information was collected from each study: primary author’s name, publication year, study type, stage of study, ancestry of participants, country where study was conducted, number of cases and controls, quality control, number of identified SNPs, chromosomal location, position, lead SNP, p-value, odds ratio (95% CI), alternate allele, alternate allele frequency, SNP location, gene(s) at locus, conflicts of interest and study limitations.

### Quality assessment and risk of bias

The strengths and weaknesses of the studies included in the systematic review were assessed following a criterion developed based on the Strengthening the Reporting of Genetic Association Studies (STREGA) statement [14], Standard Quality Assessment Criteria for Evaluating Primary Research papers and additional recommendations [15]. This criterion also assessed whether each study conducted quality control of GWAS samples, sample sizes employed, important participant characteristics and potential publication bias.

A random sample of 100 studies were independently evaluated by the three authors of this paper, following the pre-determined inclusion and exclusion criteria, with any differences in decisions resolved in a discussion afterwards. This was performed to calibrate the inclusion and exclusion judgements from all authors, in order to serve as a reference when undertaking the subsequent screening process (obtained kappa coefficient = 0.89). Furthermore, all papers associated with coronary artery disease as a reported trait in the GWAS Catalog were additionally independently evaluated by all three authors for their relevance to this study.

## Results

### Identified studies in the systematic review

Searches conducted in Embase, Medline, Global Health, Web of Science and the Cochrane Library identified a total of 3159 studies which matched the search criteria, with an additional 5 studies identified as potentially relevant through other sources, such as frequently referenced papers (total n = 3164). Papers included in the GWAS Catalog that were not identified in this search strategy were included as well in the identification process in order to capture the full known research landscape for GWAS on CAD (n = 12). Following de-duplication, a total of 2960 studies were excluded through title and abstract screening, with the primary reasons for exclusion being: not having CAD as a primary outcome of interest (n = 1465) and employing a primary methodology other than GWAS (n = 1175). After full-text screening of 114 studies following the previously stated eligibility criteria, 36 studies were selected to be included in the review [Fig 1 and Table 1 in S1 File].

### Overlap with the GWAS Catalog

The GWAS Catalog studies were independently added to the search strategy as the aim of this research was to understand what is already known in the research landscape regarding genome-wide associations between SNPs and CAD in diverse populations. As the GWAS Catalog is a credible and already well-established resource for GWA studies, an independent search in the database was deemed necessary for this research in order to ensure no known GWAS on CAD has been left out. In total, 3105 studies were identified in the independent screening of the search strategy, while 71 studies were identified from the GWAS Catalog, with there being an overlap of 59 studies between the two.

**Fig 2:**
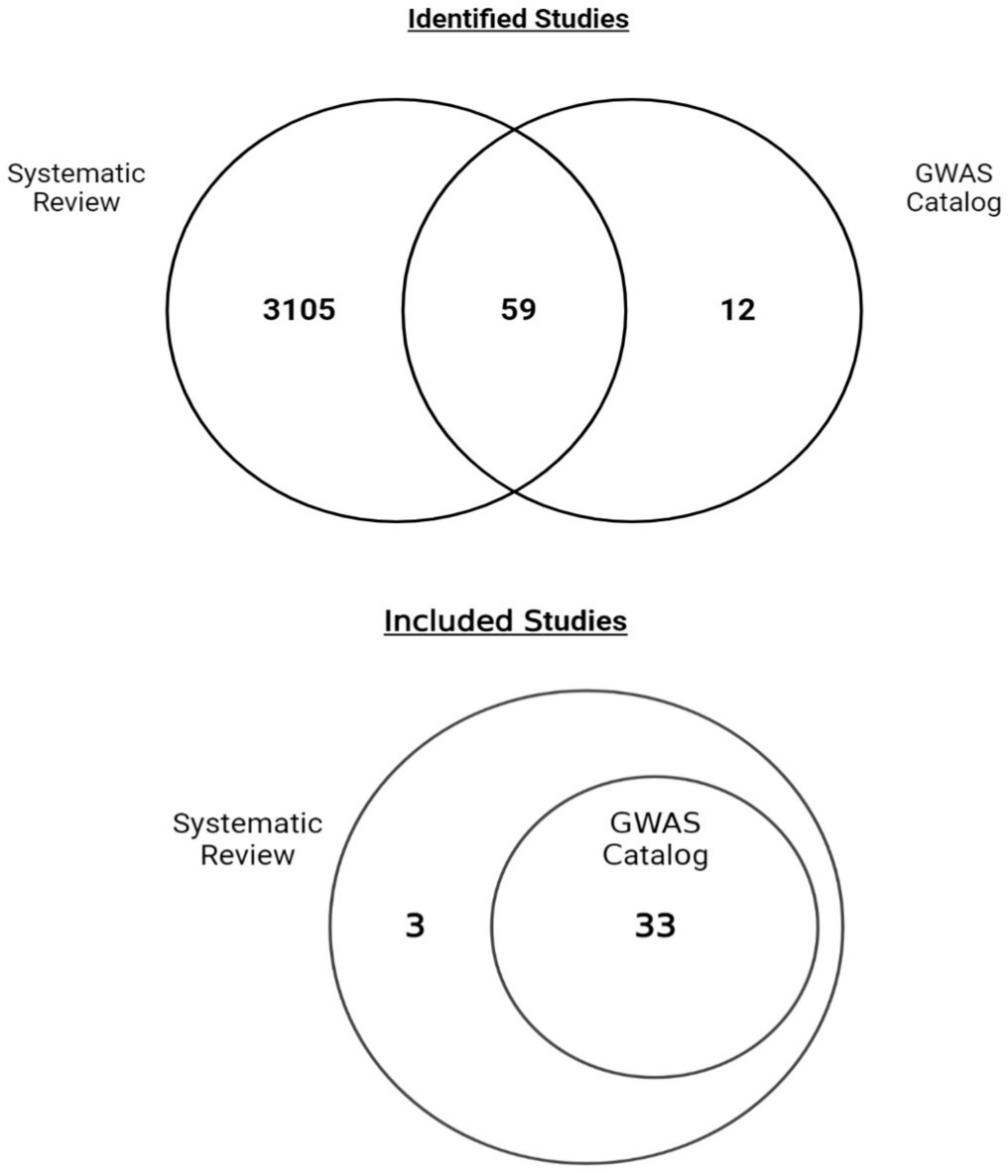
Identified and included studies for this systematic review compared to the number of coronary artery disease studies listed in the GWAS Catalog.

**Fig 3:**
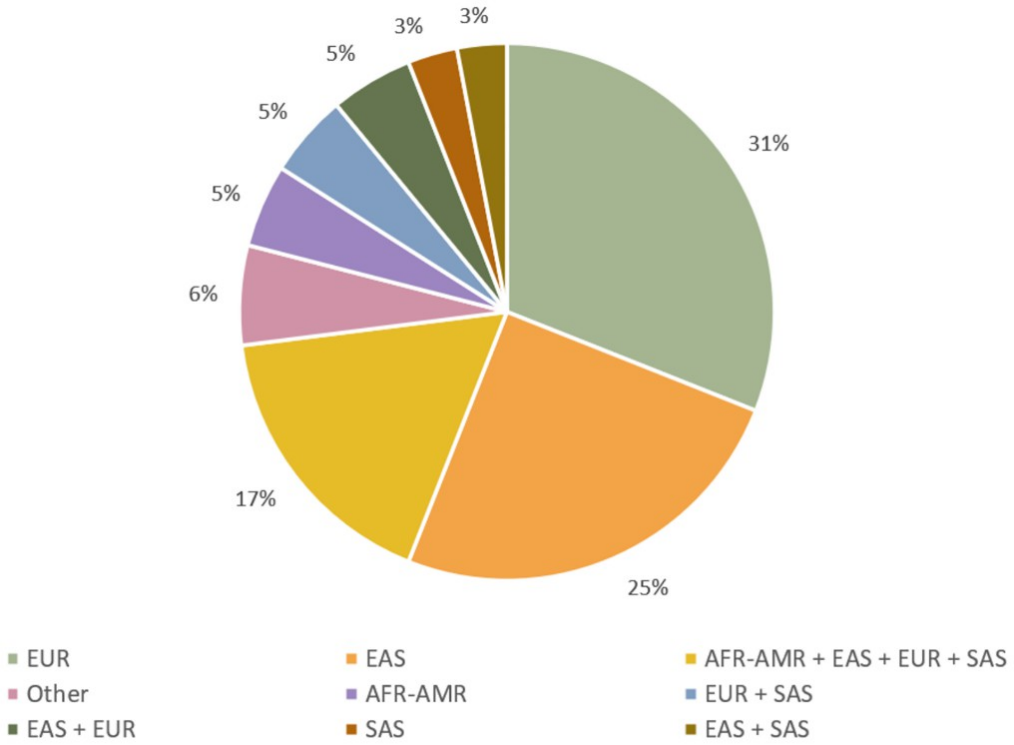
Percentage distribution of identified ancestries of participants from genome-wide association studies on coronary artery disease included in this systematic review. European (EUR); East Asian (EAS); African-American (AFR-AMR); South Asian (SAS); a combination of ancestries including those identified as African-American, Hispanic or Middle Eastern (Other).

Of the 71 listed studies in the Catalog which include “coronary artery disease” as the reported trait, 59 were also identified through the independent search strategy, with the remaining 12 studies independently included to undergo screening and eligibility assessment. Subsequent to this, 38 studies from the GWAS Catalog were excluded from this review for reasons including not conducting a GWAS, not having CAD as a primary outcome, or including participants with a diagnosed disease other than CAD. Overall, 33 studies listed in the GWAS Catalog passed eligibility screening to be included in the review [Fig 2; 3, 7, 14–48]. The full list of identified GWAS Catalog papers and eligibility decisions is shown in Table 2 in S1 File.

Three studies were identified in this review that were not included in the GWAS Catalog; a study conducted by Deloukas and colleagues in 2012, Howson and colleagues in 2017, and Verweij and colleagues in 2017.

### Identified study populations

European-ancestry individuals predominantly dominate the GWAS research landscape in the context of CAD, followed closely by East-Asian individuals and studies including a combination of African-American, East Asian, European and South Asian ancestry individuals studied together. 31% of the studies identified in this review studied only European populations, while an additional 30% studied European populations with another ancestry group. Of studies conducted in the remaining major ancestry groups worldwide, classified according to the standard established by the 100,000 Genomes Project Phase 3, it is recognized that few studies on CAD have performed GWAS in independent non-European ancestry cohorts, such as individuals of admixed American ancestry and continental African ancestry [Fig 3; 49, 50].

The majority of the included studies in this review were conducted either through a case-control study of registry data, or were meta-analyses of previously conducted case-control studies. Although studies have been identified from populations of each major ancestry group, the sample sizes vary greatly between them, with limited sample population numbers in non-European ancestry cohorts, giving rise to limitations in terms of power and generalizability of findings [Table 3 in S1 File]. Without the ability to generalize genetic findings from non-European populations across diverse populations, in addition to the already limited research into population-specific findings in the context of CAD, the ability to develop a global understanding of the genetic basis of the disease is restricted.

### Identified genetic variants

A combined total of 631 unique lead SNPs were identified from the included studies; however, it has been acknowledged that further work needs to be carried out in order to validate the findings from a number of the included papers. Approximately 75% of the studies performed two stages of the analysis (discovery and replication), while 17% performed just the discovery stage and the remaining papers were either bivariate GWAS or was a meta-analysis of other GWAS with a mix of stages included (8%).

The number of SNPs associated with CAD in more than one study was also taken into account. A total of 71 SNPs that reached genome-wide significance were identified in more than one study [S1 Table].

**Fig 4:**
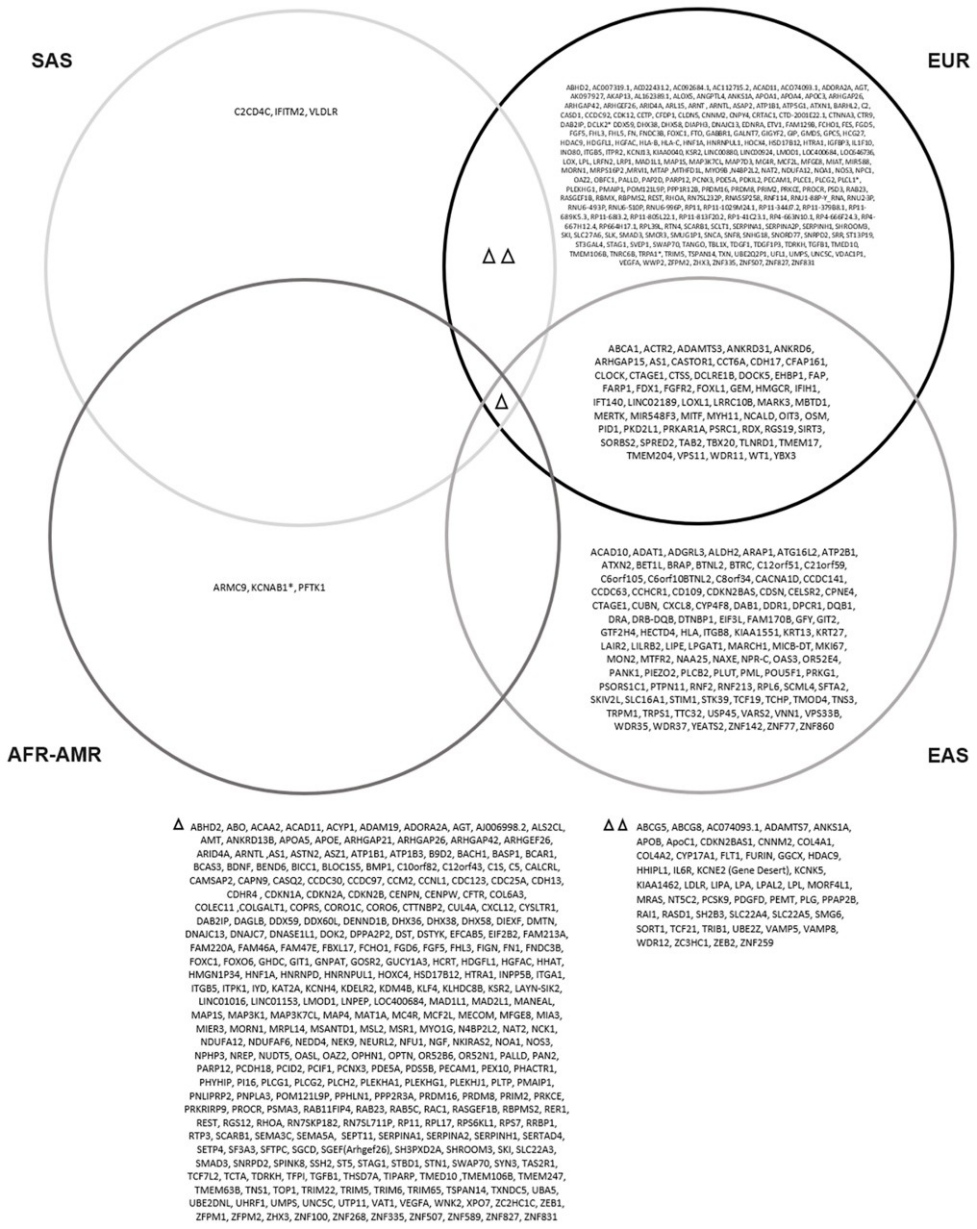
Ancestry-specific genes affected by SNPs identified in a systematic review of genome-wide association studies on coronary artery disease. African-American (AFR-AMR), European (EUR); East Asian (EAS), South Asian (SAS).

Investigation into population-specific SNPs in this systematic review shows a positive correlation between the population-representation in the research landscape and the number of population-specific SNPs identified to be associated with CAD [Figs 3 and 4]. This may be due to the fact that gene panels employed for GWAS have been developed with a bias towards individuals of European ancestry and therefore may overlook candidate risk genes in populations of other ancestries [1, 7, 51].

## Discussion

In this review, including a total of 743,919 CAD case participants from 25 different countries, 631 unique genetic loci have been identified to be associated with CAD. Despite representing participants from a number of major ancestry groupings, the distribution of studies included in this review remains reflective of the current study landscape, with only approximately 42% of the studies included in this review including participants of non-European ancestry. This underrepresentation of non-European individuals in genetic studies, specifically indigenous groups, admixed Americans, South Asians living in Asia and populations of African ancestry who are living in continental Africa, results in many conclusions regarding what we understand about SNP-trait associations to be drawn from a biased ancestry cohort.

Being a prominent non-communicable disease worldwide, CAD is an extensively studied disease with many well-established understandings in place regarding both the environmental and genetic contributions towards its development, as evidenced by the number of studies identified in this review. This research, although rich and valuable, does not cover the entirety of the disease, as the majority our understandings have been drawn from a single ancestry group. It remains elusive as to whether or not different genes are contributing towards the pathophysiology of the disease in populations of different ancestry, or whether the effect sizes of each gene are what differs between them; however, this provides evidence for gaps in the literature to venture into this research and contribute towards this understanding of CAD as a global disease.

### Systematic review

In this research, SNPs which were identified to be associated with CAD in different identified papers included only one or two studies pertaining to different ancestry subgroups, which was too small a number to carry out a meta-analysis within each ancestry group. Furthermore, a meta-analysis was not possible as many of the studies employing diverse-ancestry cohorts did not present subgroup specific results for the GWAS, meaning identifying ancestry-specific genetic loci associated with CAD in these studies was not possible. More large-scale GWAS studies in diverse populations are necessary if we are to be able to compare genetic loci between ancestries and subsequently draw conclusions with significant power [52, 53]. With larger sample sizes, the characterization of functional alleles and their respective associations with CAD can be elucidated.

Additionally, the large number of SNPs identified in ancestry-specific cohorts such as the East Asian ancestry group is indicative of the value of conducting research in more diverse settings. Having a different subset of genes pertaining to the relationship between a specific ancestry group and CAD contributes towards the understanding the variety of genetic architecture of CAD across populations [7, 9, 21].

With an increasing number of studies beginning to identify novel population-specific CAD loci, resulting findings can be added to the GWAS reference panel in order to capture a more accurate representation of the global population. This highlights the importance of diversity in GWAS analyses, as although heritability estimates may differ both between and within ancestry populations as a result of variation in environmental and population genetic factors, capturing a more diverse population reference group overall will expectantly yield novel opportunities for discovery and validation of GWAS findings, while contributing towards the understanding of CAD as a whole.

While it is important to understand the differences between diverse populations, the identification of shared findings between populations offer equally valuable insights into understanding the biological mechanisms underlying a disease. In this systematic review, 71 SNPs were identified in more than one study from populations of different ancestries. Shared findings across diverse populations can be used for further research into the causational relationship between the identified shared SNPs and CAD through means of mendelian randomization analysis, and also lends itself towards the investigation of the functional annotation of the identified variants and biological pathway analysis which is necessary for developing a more global understanding of the pathophysiology of CAD. The identification of both shared and population-specific SNPs in this review supports the need to develop genetic research more representative of diverse ancestry populations, in an effort to understand CAD from a perspective which ensures underrepresented populations can also benefit from research relevant to their health [Fig 4].

### GWAS Catalog

While well-established curated databases of GWAS do exist, with an influx of new research, especially regarding the identification and comparisons of genetic findings between diverse populations, it was forecasted prior to this review that some relevant studies may not have been included in databases such as the GWAS Catalog. It was found that three studies identified to be relevant to the research objective in this paper were not identified in the GWAS Catalog [17, 37, 43].

From the onset of conducting this research, many searches were run against findings from the GWAS Catalog in order to keep up to date with the weekly release of data [13, 50]. The data for this systematic review and GWAS Catalog extraction were obtained on the 6^th^ of October and 10^th^ of October 2022 respectively. This is important to note, as the ever-changing research landscape will expectantly change the number of identified studies for this topic when run in the future, and the GWAS Catalog will continue to update its database whenever relevant papers are identified.

The three papers not included in the GWAS Catalog are evidence of the recommendation that researchers wanting to find relevant GWAS papers on a specific trait of interest should not rely solely on consortiums such as the GWAS Catalog for information, and instead use it as a reference while conducting an independent search in order to not potentially overlook anything relevant to the research.

Our findings regarding the observed lag from the Catalog in terms of including all relevant studies have been raised with the Catalog directly by the author on this paper, Segun Fatumo. He additionally raised the point that there is a lack of included GWA studies from the African subcontinent despite the research and findings from such studies being published. Although this was not addressed in the context of CAD, it reinforces the conclusion that, irrespective of the trait of interest, an independent search is still required if we are to truly capture the relevant research landscape of GWAS.

### Implications for populations of different ancestries

The findings of both shared and ancestry-specific SNPs across diverse ancestry populations are vital in contributing towards our ability to characterize CAD in under-represented populations. The identification of ancestry-specific SNPs provides insight into the existence of unique ancestry-specific genetic risk factors, disease mechanisms and pathways relating to CAD. Although limited by the scope of the current research landscape, the inclusion of diverse ancestries in CAD research will expectantly provide new perspectives into the biological mechanisms and genetic pathways relating to the disease, as evidenced by the ancestry-specific findings in East Asian populations [Fig 4]. It is already known that CAD is a complex disease which is heavily influenced by the combination of both genetic and environmental factors. With the identification of both shared and ancestry-specific genetic risk factors alongside this, a more comprehensive understanding of CAD can be developed, which is vital towards developing more precision-based treatments and targeted interventions for individuals in under-represented populations. Closing the representation gap of non-European ancestry populations in research on CAD is essential for addressing known health disparities and disease risks, while also ensuring advancements of research into CAD can be beneficial for more diverse global populations.

A notable gap in the research relates to the absence of African populations from continental Africa in the research landscape. African populations are known to have the most genetic diversity in the world, and by under-representing the entire continent when conducting genetic research, we overlook invaluable opportunities to understand genetic pathways and adaptations which we otherwise wouldn’t see globally. The absence of representation of populations from continental Africa in the research landscape amplifies the potential for observed health disparities in these populations, as current healthcare policies, interventions and drug treatments would have been developed based on research which overlooked the unique genetic diversity of African populations. This has the potential to lead to misdiagnosis, inadequate risk management, and ineffective treatments for individuals of African descent, specifically populations in continental Africa.

This gap in the ability to develop a comprehensive understanding of diseases is also true for South Asian populations and other populations of non-European ancestry which are underrepresented in research. The identification of ancestry-specific SNPs serves as a starting point for engaging under-represented populations in research. By prioritizing increasing representation and diversity in genetic research through building trust within communities and providing tailored healthcare from research relevant to their health, more effective treatment outcomes can be developed and healthcare disparities between populations can begin to be addressed as well.

### Strengths and limitations

This study is not without limitations. Firstly, the accuracy in which the developed search strategy identified papers of interest was conflicted by the selection of search terms. Terms relating to “ancestry” were purposefully excluded from the search string in order to minimize the potential loss of papers due to different definitions of study populations. On the other hand, some of the selected terms employed in the developed search string may have narrowed the results to the point that important papers were overlooked; the inclusion of broader search terms relating to “GWAS” specifically would expectedly be beneficial to identify a wider search area. Alongside this, the strict eligibility criteria for this search strategy may have additionally missed or excluded potentially relevant studies. Only search terms related to “GWAS” and “coronary artery disease” in the title or abstract of the paper were included; therefore, due to varying definitions of the disease, it is possible that some papers which used alternative words to describe either, such as “myocardial infarction”, may have been overlooked. Another limitation to consider is how studies each defined and differentiated ancestry, ethnicity and race; ethnicity and race are regarded as more of a cultural and social identity as opposed to ancestry which relates to one’s genetic heritage. It is possible that for some of the included studies, some participants’ genetic ancestry and cultural ethnicity were not aligned and therefore could have been included in a population group which was their reported ethnicity instead of ancestry.

Furthermore, only journal articles were included in this review, which additionally could have limited the number and significance of findings. Although efforts were made to identify and include all relevant studies, the possibility of publication bias should not be overlooked. However, we were reassured to see that we identified all studies that were relevant from the GWAS Catalog.

A strength of the study is that we used two approaches- a systematic review and the GWAS Catalog to ensure that we identify all relevant studies.

To the best of our knowledge, to date, no large-scale genetic association study has been carried out that primarily focuses on comparing the differences in genetic architecture of CAD across major ancestry groups. The systematic review study design supplements such a study until more research is carried out as it lends itself towards generating a more representative sample of participants in order to identify both novel and shared patterns of genetic association of CAD pathophysiology both within and between different population groups.

## Conclusion

This systematic review summarized what is currently known with regards to GWAS findings of CAD in diverse ancestry populations. We were able to highlight the existence of both shared and ancestry-specific genetic factors underlying CAD, and brought to attention the lack of representation of non-European ancestry populations in the research landscape. Significant gaps exist in the representation of both South Asian populations and populations from continental Africa, with research from both population groups likely allowing for a more complete understanding of disease mechanisms and pathophysiology in the future. As more GWAS on CAD begin to be carried out, it is with hope that the inclusion of greater sample sizes representative of the global population will allow for better interrogation and extrapolation of disease-specific genetic findings.

## Supporting information

S1 File (XLSX)

S1 Table SNPs associated with CAD in more than one study. (PDF)

S1 Checklist PRISMA 2020 checklist. (DOCX)

## Abbreviations

AFR-AMR: African-American
CAD: Coronary artery disease
CI: Confidence interval
EAS: East Asian
EUR: European
GWAS: Genome-wide association study
PRISMA: Preferred Reporting Items for Systematic Reviews and Meta-Analyses
PROSPERO: Prospective Register of Systematic Reviews
SAS: South Asian
SNP: Single-nucleotide polymorphism
