# Supplementary material for: Genome-wide association studies on coronary artery disease: A systematic review and implications for populations of different ancestries
Source: PLoS One. 2023 Nov 29;18(11):e0294341. doi: 10.1371/journal.pone.0294341 (PMC10686512; doi:10.1371/journal.pone.0294341)
Supplement: S1 Table — (PDF) [file pone.0294341.s002.pdf]

Table 1. SNPs associated with CAD in more than one study

Reference	Chromosome	Lead SNP	P-Value	Gene(s) at Locus	Ancestry
Samani et al., 2007	1p13.3	rs599839	4.05E-09	PSRC1	EUR
Yamada et al., 2018			3.52E-05		EAS
Deloukas et al., 2012	1p13.3	rs602633	1.47E-25	SORT1	EUR & SAS
Yamada et al., 2018			1.15E-05	N/A	EAS
Deloukas et al., 2012	1p32.2	rs17114036	5.80E-12	PPAP2B	EUR & SAS
Schunkert et al., 2011			3.81E-19		EUR
Deloukas et al., 2012	1p32.3	rs11206510	1.79E-05	PCSK9	EUR & SAS
Tcheandjieu et al., 2022			3.20E-07		EUR
Tcheandjieu et al., 2022	1p34.3	rs61776719	1.07E-06	RNU6-510P-FHL3	EUR
van der Harst et al., 2018			1.10E-09	FHL3, UTP11, SF3A3, MANEAL, INPP5B	AFR + EAS + EUR + SAS
Tcheandjieu et al., 2022	1p36.32	rs2493298	1.85E-08	PRDM16	EUR
van der Harst et al., 2018			1.90E-09	PRDM16, PEX10, PLCH2, RER1	AFR + EAS + EUR + SAS
Nelson et al., 2017	1q21.3	rs11810571	4.24E-08	TDRKH	AFR + EAS + EUR + SAS
Verweij et al., 2017			1.72E-10		AFR + EAS + EUR + SAS
Howson et al., 2017	1q32.1	rs6700559	1.13E-08	DDX59–CAMSAP2	AFR + EAS + EUR + SAS
Tcheandjieu et al., 2022			1.19E-05		EUR
Deloukas et al., 2012	2p21	rs6544713	2.12E-09	ABCG5-ABCG8	EUR & SAS
Tcheandjieu et al., 2022			2.22E-10		EUR
Deloukas et al., 2012	2p24.1	rs515135	2.56E-10	APOB	EUR & SAS
Tcheandjieu et al., 2022			4.88E-08		EUR
Deloukas et al., 2012	2q33.2	rs6725887	1.16E-15	WDR12	EUR & SAS
Tcheandjieu et al., 2022			5.47E-18		EUR
Howson et al., 2017	2q35	rs2571445	4.55E-10	TNS1	AFR + EAS + EUR + SAS
Tcheandjieu et al., 2022			1.88E-08		EUR
Klarin et al., 2017	3p21.31	rs7623687	2.00E-08	RHOA	EUR

Nelson et al., 2017			3.44E-10		AFR + EAS + EUR + SAS
Verweij et al., 2017			3.28E-08	RHOA, AMT, TCTA, CDHR4 , KLHDC8B	AFR + EAS + EUR + SAS
Tcheandjieu et al., 2022	3q21.2	rs142695226	6.19E-05	UMPS–ITGB5	EUR
Nelson et al., 2017			1.53E-09		AFR + EAS + EUR + SAS
Verweij et al., 2017			1.70E-10		AFR + EAS + EUR + SAS
Tcheandjieu et al., 2022	3q22.3	rs667920	1.14E-05	STAG1	EUR
van der Harst et al., 2018			6.00E-15	STAG1, MSL2, NCK1, PPP2R3A	AFR + EAS + EUR + SAS
Deloukas et al., 2012	3q22.3	rs9818870	2.62E-09	MRAS	EUR & SAS
Erdmann et al., 2009			7.44E-13		EUR
Tcheandjieu et al., 2022			1.67E-07		EUR
Klarin et al., 2017	3q25.2	rs12493885	1.02E-09	ARHGEF26	EUR
Nelson et al., 2017			3.16E-08		AFR + EAS + EUR + SAS
Nikpay et al., 2015	4q12	rs17087335	4.60E-08	REST-NOA1	AFR + EAS + EUR + SAS
Tcheandjieu et al., 2022			4.91E-05		EUR
Klarin et al., 2017	4q21.21	rs10857147	3.39E-08	FGF5	EUR
Nelson et al., 2017			5.66E-09	PRDM8–FGF5	AFR + EAS + EUR + SAS
Verweij et al., 2017			4.29E-10		AFR + EAS + EUR + SAS
Tcheandjieu et al., 2022	4q32.3	rs7696431	3.17E-06	PALLD	EUR
van der Harst et al., 2018			2.70E-08	PALLD, DDX60L	AFR + EAS + EUR + SAS
Tcheandjieu et al., 2022	5p15.31	rs1508798	1.10E-06	SNHG18-CTD-2001E22.1	EUR
van der Harst et al., 2018			4.80E-13	SEMA5A, TAS2R1	AFR + EAS + EUR + SAS
Tcheandjieu et al., 2022	6p11.2	rs9367716	2.83E-07	RAB23-PRIM2	EUR
van der Harst et al., 2018			9.60E-10	PRIM2, RAB23, DST, BEND6	AFR + EAS + EUR + SAS
Nelson et al., 2017	6p22.3	rs6909752	2.19E-09	HDGFL1	AFR + EAS + EUR + SAS
Tcheandjieu et al., 2022			8.06E-07		EUR
Hager et al., 2012	6p24.1	rs9349379	8.02E-10	PHACTR1	Middle Eastern
Tcheandjieu et al., 2022			1.01E-41		EUR

Yamada et al., 2018			1.69E-04		EAS
Deloukas et al., 2012	6p24.1	rs9369640	7.53E-22	PHACTR1	EUR & SAS
Yamada et al., 2018			1.30E-04		EAS
Deloukas et al., 2012	6q23.2	rs12190287	4.94E-13	TCF21	EUR & SAS
Schunkert et al., 2011			1.07E-12		EUR
Tcheandjieu et al., 2022	7p13	rs2107732	4.33E-06	CCM2	EUR
van der Harst et al., 2018			3.60E-08	CCM2, MYO1G	AFR + EAS + EUR + SAS
Deloukas et al., 2012	7q32.2	rs11556924	6.74E-17	ZC3HC1	EUR & SAS
Schunkert et al., 2011			9.18E-18		EUR
Tcheandjieu et al., 2022			4.76E-14		EUR
Nikpay et al., 2015	7q36.1	rs3918226	1.70E-09	NOS3	AFR + EAS + EUR + SAS
Tcheandjieu et al., 2022			6.14E-08		EUR
Tcheandjieu et al., 2022	8p21.3	rs6984210	3.07E-07	BMP1	EUR
van der Harst et al., 2018			2.10E-11	BMP1, SFTPC, DMTN, PHYHIP, DOK2, XPO7	AFR + EAS + EUR + SAS
Deloukas et al., 2012	8p21.3	rs264	2.88E-09	LPL	EUR & SAS
Tcheandjieu et al., 2022			3.17E-07		EUR
Tcheandjieu et al., 2022		rs2342572	2.02E-08	SETP4-UBE2DNL	AMR + AFR + EAS + EUR
Koyama et al., 2020	8q13.2		2.50E-08	C8orf34	EAS
Deloukas et al., 2012	8q24.13	rs2954029	4.75E-09	TRIB1	EUR & SAS
Tcheandjieu et al., 2022			8.94E-11		EUR
Takeuchi et al., 2012	9p21	rs671	6.10E-16	ALDH2	EAS
Yamada et al., 2018	12q24.12		4.12E-15		EAS
Tcheandjieu et al., 2022	9p21	rs2891168	1.91E-107	CDKN2BAS	EUR
Lee et al., 2013	9p21.3		1.83E-08		EAS
Lee et al., 2013	9p21.3	rs4977574	1.36E-08	CDKN2BAS	EAS
Yamada et al., 2018			4.18E-06	CDKN2B-AS1	EAS
Deloukas et al., 2012	9p21.3	rs1333049	1.39E-52	CDKN2BAS1	EUR & SAS

Guo et al., 2018			1.00E-25	CDKN2BAS	EAS
Lee et al., 2013			4.43E-09	N/A	EAS
Samani et al., 2007			6.12E-05	N/A	EUR
Wild et al., 2011			7.12E-58	N/A	EUR
Yamada et al., 2018			3.95E-06	N/A	EAS
Koyama et al., 2020	9q31.1	rs35093463	3.50E-09	ABCA1	EAS
Koyama et al., 2020			1.10E-02		EAS & EUR
Tcheandjieu et al., 2022	9q31.2	rs944172	2.19E-05	RNU6-996P-AL162389.1	EUR
van der Harst et al., 2018			1.10E-11	KLF4	AFR + EAS + EUR + SAS
Deloukas et al., 2012	9q34.2	rs579459	2.66E-08	ABO	EUR & SAS
Schunkert et al., 2011			4.08E-14		EUR
Peden et al., 2011	10p11	rs2505083	3.87E-08	KIAA1462	EUR & SAS
Deloukas et al., 2012	10p11.23		1.35E-11		EUR & SAS
Tcheandjieu et al., 2022	10p13	rs61848342	7.13E-05	RN7SL232P	EUR
van der Harst et al., 2018			6.30E-10	CDC123, NUDT5, OPTN	AFR + EAS + EUR + SAS
Deloukas et al., 2012	10q11.21	rs501120	1.79E-09	CXCL12	EUR & SAS
Samani et al., 2007			9.46E-08		EUR
Peden et al., 2011	10q23	rs1412444	2.76E-13	LIPA	EUR & SAS
Wild et al., 2011			3.71E-08		EUR
Tcheandjieu et al., 2022	10q23.1	rs17680741	4.25E-06	TSPAN14	EUR
van der Harst et al., 2018			2.30E-11	TSPAN14, MAT1A, FAM213A	AFR + EAS + EUR + SAS
Tcheandjieu et al., 2022	10q23.31	rs2246833	1.10E-13	LIPA	EUR
Deloukas et al., 2012			9.49E-06		EUR & SAS
Wild et al., 2011			4.35E-08		EUR
Deloukas et al., 2012	10q24.32	rs12413409	6.26E-08	CYP17A1-CNNM2-NT5C2	EUR & SAS
Schunkert et al., 2011			1.03E-09		EUR
Tcheandjieu et al., 2022	10q24.33	rs4918072	5.03E-06	OBFC1-SLK	EUR

van der Harst et al., 2018			2.60E-09	STN1, SH3PXD2A	AFR + EAS + EUR + SAS
Tcheandjieu et al., 2022	11p11.2	rs7116641	3.23E-05	HSD17B12	EUR
van der Harst et al., 2018			1.00E-08		AFR + EAS + EUR + SAS
Nelson et al., 2017	11p15.3	rs3993105	4.77E-08	ARNTL	AFR + EAS + EUR + SAS
Tcheandjieu et al., 2022			6.57E-05		EUR
Schunkert et al., 2011	11q23.3	rs964184	1.02E-17	ZNF259, APOA5-A4-C3-A1	EUR
Tcheandjieu et al., 2022			3.68E-14	ZNF259-APOA5-APOA1	EUR
Klarin et al., 2017	12p12.2	rs10841443	2.23E-08	RP11-664H17.1	EUR
Tcheandjieu et al., 2022			3.24E-06	RP664H17.1	EUR
Tcheandjieu et al., 2022	12q13.13	rs11170820	2.01E-07	HOCX4	EUR
Verweij et al., 2017			4.09E-08		AFR + EAS + EUR + SAS
Lee et al., 2013	12q24	rs11066015	4.51E-11	ACAD10	EAS
Yamada et al., 2018	12q24.12		4.92E-15		EAS
Deloukas et al., 2012	12q24.12	rs3184504	5.44E-11	SH2B3	EUR & SAS
Tcheandjieu et al., 2022			1.00E-07		EUR
Yamada et al., 2018	12q24.12	rs3782886	4.38E-13	BRAP	EAS
Takeuchi et al., 2012	12q24.23		1.60E-34		EAS
Howson et al., 2017	12q24.31	rs11057830	1.34E-08	SCARB1	AFR + EAS + EUR + SAS
Tcheandjieu et al., 2022			1.26E-12		EUR
Klarin et al., 2017	12q24.31	rs2244608	2.41E-08	HNF1A	EUR
Nelson et al., 2017			7.74E-10		AFR + EAS + EUR + SAS
Tcheandjieu et al., 2022			3.68E-06		EUR
Verweij et al., 2017			1.86E-10	HNF1A, OASL	AFR + EAS + EUR + SAS
Deloukas et al., 2012	13q34	rs4773144	1.43E-11	COL4A1-COL4A2	EUR & SAS
Schunkert et al., 2011			3.84E-09		EUR
Tcheandjieu et al., 2022	14q32.13	rs112635299	2.61E-09	SERPINA2P-SERPINA1	EUR
van der Harst et al., 2018			8.40E-10		AFR + EAS + EUR + SAS

Deloukas et al., 2012	14q32.2	rs2895811	4.08E-10	HHIPL1	EUR & SAS
Schunkert et al., 2011			1.14E-10		EUR
Nikpay et al., 2015	15q22.33	rs56062135	4.50E-09	SMAD3	AFR + EAS + EUR + SAS
Tcheandjieu et al., 2022			1.98E-12		EUR
Deloukas et al., 2012	15q26.1	rs17514846	9.33E-11	FURIN-FES	EUR & SAS
Tcheandjieu et al., 2022			4.42E-08		EUR
Klarin et al., 2017	16q23.1	rs3851738	2.43E-08	CFDP1	EUR
Tcheandjieu et al., 2022			1.74E-10		EUR
Klarin et al., 2017	16q23.3	rs7500448	1.20E-08	CDH13	EUR
Nelson et al., 2017			4.76E-10		AFR + EAS + EUR + SAS
Tcheandjieu et al., 2022			1.33E-11		EUR
Verweij et al., 2017			4.83E-11		AFR + EAS + EUR + SAS
Deloukas et al., 2012	17p11.2	rs12936587	1.24E-09	RAI1-PEMT-RASD1	EUR & SAS
Schunkert et al., 2011			4.45E-10	RASD1, SMCR3, PEMT	EUR
Schunkert et al., 2011	17p13.3	rs216172	1.15E-09	SMG6, SRR	EUR
Tcheandjieu et al., 2022			1.18E-06	SMG6	EUR
Howson et al., 2017	17q21.32	rs17608766	2.10E-07	GOSR2	AFR + EAS + EUR + SAS
Tcheandjieu et al., 2022			2.51E-05		EUR
Howson et al., 2017	17q23.3	rs1867624	3.98E-08	PECAM1	AFR + EAS + EUR + SAS
Tcheandjieu et al., 2022			3.85E-10		EUR
Klarin et al., 2017	19q13.2	rs8108632	2.35E-08	TGFB1	EUR
Nelson et al., 2017			4.04E-08		AFR + EAS + EUR + SAS
Tcheandjieu et al., 2022			2.10E-07		EUR
Verweij et al., 2017			9.54E-09	TGFB1, B9D2	AFR + EAS + EUR + SAS
Tcheandjieu et al., 2022	20q13.12	rs3827066	2.62E-09	ZNF335	EUR
van der Harst et al., 2018			4.40E-09	PCIF1, ZNF335, NEURL2, PLTP	AFR + EAS + EUR + SAS

Ancestry classifications followed the standard of the 10,000 Genome Project; AMR = Admixed American, AFR = African, EAS = South Asian, EUR = European, and SAS = South Asian. Any identified ancestry populations that were unable to be classified into one of these categories have been listed in their full name.
